# Continuous Light Does Not Compromise Growth and Yield in Mini-Cucumber Greenhouse Production with Supplemental LED Light

**DOI:** 10.3390/plants10020378

**Published:** 2021-02-17

**Authors:** Jason Lanoue, Jingming Zheng, Celeste Little, Bernard Grodzinski, Xiuming Hao

**Affiliations:** 1 Harrow Research and Development Centre, Agriculture & Agri-Food Canada, Harrow, ON N0R 1G0, Canada; Jason.Lanoue@canada.ca (J.L.); Jingming.Zheng@canada.ca (J.Z.); Celeste.Little@canada.ca (C.L.); 2 Department of Plant Agriculture, University of Guelph, Guelph, ON N1G 2W1, Canada; bgrodzin@uoguelph.ca

**Keywords:** photoperiod, light-emitting diode, greenhouse, photosynthesis, cucumber, yield, continuous lighting, economical analysis

## Abstract

Continuous lighting (CL, 24 h) can reduce the light intensity/light capital costs used to achieve the desired amount of light for year-round greenhouse vegetable production in comparison to short photoperiods of lighting. However, growth under CL has led to leaf injury characterized by chlorosis unless a thermoperiod or alternating light spectrum during CL is used. To date, there is no literature relating to how cucumbers (*Cucumis* *sativus*) respond to CL with LEDs in a full production cycle. Here, we evaluated a mini-cucumber cv. “Bonwell” grown under 4 supplemental lighting strategies: Treatment 1 (T1, the control) was 16 h of combined red light and blue light followed by 8 h of darkness. Treatment 2 (T2) had continuous (24 h) red light and blue light. Treatment 3 (T3) was 16 h of red light followed by 8 h of blue light. Treatment 4 (T4) was 12 h of red light followed by 12 h of blue light. All treatments had a supplemental daily light integral (DLI) of ~10 mol m^−2^ d^−1^. Plants from all treatments showed similar growth characteristics throughout the production cycle. However, plants grown under all three CL treatments had higher chlorophyll concentrations from leaves at the top of the canopy when compared to T1. The overall photosynthetic capacity, light use efficiency, and photosynthetic parameters related to light response curves (i.e., dark respiration, light compensation point, quantum yield, and photosynthetic maximum), as well as the quantum yield of photosystem II (PSII; F_v_/F_m_) were similar among the treatments. Plants grown under all CL treatments produced a similar yield compared to the control treatment (T1). These results indicate that mini-cucumber cv. “Bonwell” is tolerant to CL, and CL is a viable and economical lighting strategy for mini-cucumber production.

## 1. Introduction

Artificial light is needed to supplement sunlight during the light limiting winter months for year-round production to meet consumer demand for fresh vegetables in regions with low sunlight. The yield and plant biomass increases from supplemental light are largely determined by daily light integral (DLI, light intensity x photoperiod) received by the plants. Long photoperiods of lighting have an economic advantage over a short period of lighting in achieving the same DLI with less light fixtures (capital) costs. More recently, the trend towards continuous (24 h) supplemental lighting has received much interest due to the potential for increased yield and its inherent reduction in energy input [1,2,3,4]. The use of continuous light (CL) theoretically can increase the production as there is constant light energy to drive photosynthesis and carbon assimilation [5]. However, to date, the hypothetical increase in growth has not been realized in large vining crops such as cucumber, tomato, and pepper, which in Canada account for 97% of greenhouse production (approximately 4200 acres). This is mostly due to the negative effects of CL, such as leaf chlorosis, reducing the plants’ ability to utilize the added radiation [6].

Most of what we know about the effects of CL on vascular plant growth comes from research on tomatoes. Hypotheses ranging from circadian asynchrony [7] to improper carbohydrate metabolism [2,8] to the effects on the light-harvesting complex [9] have been explored to explain why CL causes the injury. Having both CL sensitive and CL insensitive accessions, studies on tomatoes have narrowed in on the expression of a key light-harvesting complex protein (*CAB-13*) as potentially the ultimate controller of CL sensitivity [9]. The ability to adequately express *CAB-13* throughout a CL period may allow for the hypothesized increase in plant production. More recently, a greenhouse trial using an alternating red and blue light-emitting diode (LED) CL strategy was the first of its kind to show that tomatoes can be grown for a full production season without showing the typical signs of chlorosis associated with CL-injury [4]. This, along with other studies investigating the role of photoreceptors [10], has raised the question of what role spectral quality can play in CL tolerance and the adoption of CL strategies for production.

Cucumbers, like tomatoes, are a popular greenhouse crop grown throughout the world. The practice of supplemental lighting has been commonplace during cucumber production for decades [11]. Typical photoperiods for cucumber production are between 18 and 20 h [12,13,14]. However, similar to studies on tomatoes, greenhouse-grown cucumbers under supplemental CL provided by high-pressure sodium (HPS) showed mild and severe leaf chlorosis (characteristic of CL-injury) at four and nine weeks into the treatment, respectively [15]. The severe leaf chlorosis observed 9 weeks into the treatment was coupled with a 35% decrease in fruit yield [15]. Furthermore, a decrease in leaf starch levels was also observed, indicating that growth under CL inhibits proper gas exchange and carbohydrate assimilation [15].

Shibaeva and Markovskaya [16] examined cucumber plants grown in growth chambers under high-pressure mercury lamps at different light intensities, photoperiod lengths, and plant ages. It was found that at 14 days into the treatment, leaves grown under all light intensities (60, 120, and 160 µmol m^−2^ s^−1^) and photoperiod lengths (16 h, 20 h, and 24 h) produced the same quantum efficiency of PSII (F_v_/F_m_, a normalized ratio between variable fluorescence by maximum fluorescence), a parameter often used to convey the health of leaves [17]. However, at 21–28 days, cucumber leaves at 120 and 160 µmol m^−2^ s^−1^ had lower F_v_/F_m_ values under CL compared to the 16 h and 20 h photoperiods [16]. These results indicate that similar to tomato plants grown under CL longer than 1 week, injury is observed, which may then reduce plant performance and ultimately yield.

Similar to tomatoes, LEDs are being adopted for cucumber production and have shown increases in yield and reductions in energy use compared to the traditional HPS system [18,19]. However, the use of CL during cucumber production has still proven to be elusive thus far. It has been stated that “cucumbers need a daily dark period in order to achieve optimal growth [19]” and that “continuous illumination should not be implemented in commercial cucumber production [15]”. This is due both to the decrease in vegetative growth [20] and yield reduction [15], which occurred when cucumbers were grown under CL. However, based on our recent finding using dynamic spectral changes to eliminate leaf injury on tomatoes [4], it may be feasible to overcome CL-related injury in greenhouse cucumbers. Therefore, we set out to examine the morphological, physiological, and yield characteristics of mini-cucumber production during CL with wavelength-specific LEDs and its potential as a viable production strategy. It is hypothesized that, similar to tomatoes, the use of timely wavelength-specific LEDs can mitigate the injury observed from plants grown under CL with HPS lighting. Further, we provide an economic analysis comparing the three CL strategies used in this study to a traditional 16 h photoperiod.

## 2. Materials and Methods

### 2.1. Plant Material and Experimental Design

Mini-cucumber seeds cv. “Bonwell” (Rijk Zwaan; De Lier, Netherlands) were sown onto Rockwool plugs on 9 October 2019, and placed in a glass greenhouse for germination. On 29 October, the cucumber transplants were planted onto Rockwool slabs on top of raised growing troughs (60 cm high) in a large glass greenhouse (200 m^2^ growing area) at the Harrow Research and Development Center (Agriculture and Agri-Food Canada, Harrow, ON, Canada; 42.03° N, 82.9° W) at a plant density of 2.74 plants m^−2^. Plants were trained into a “V” high wire growth system. Once plant heads reached the overhead wires (3 m), bottom leaves were removed, and the plants were lowered as needed. The plants were drip-irrigated using a complete nutrient solution (Ontario Ministry of Agriculture, Food and Rural Affairs (OMAFRA), 2010). The electrical conductivity and pH were set at 2.8 dS m^−1^ and 5.8, respectively. The greenhouse was enriched to a 1000 µL L^−1^ CO_2_ concentration during both day and night when it was not ventilated. The day/night heating temperature setpoints were 22 °C/20 °C for the first 2 weeks after planting to promote vegetative growth and then reduced to 21 °C/19 °C for the rest of the growth period to maintain the balance of vegetative and generative growth. Day/night ventilation temperature setpoints were 25 °C/22 °C. Using a higher ventilation temperature allows the greenhouse’s actual temperature to naturally increase with strong solar radiation without additional use of energy and improves plant growth because the optimal growth temperature increases with light intensity. The humidity was maintained at 70 ± 10%. Temperature setpoints were relatively similar between day and night as T2 has relatively high nighttime light intensity and thus required a higher than normal nighttime temperature. It is also for reducing internode length to reduce the labor requirement in lowering the cucumber plants.

**Figure 2:**
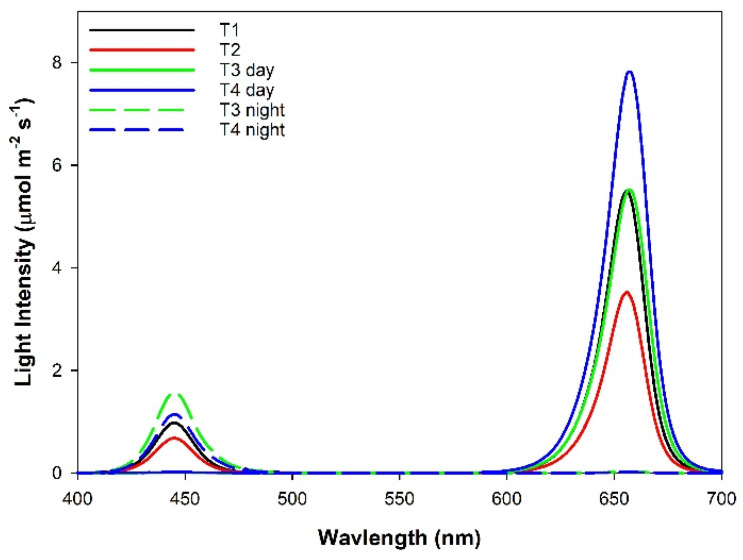
Spectral composition of light treatments as determined with a Li-180 spectroradiometer during the night.

The glass greenhouse was divided into six rows, with those on the perimeter of the greenhouse serving as guard rows. The four inside rows were separated by reflective curtains, which were impenetrable to light. Each row was further divided into four lighting plots, each containing 16 plants. There was a 1.7 m gap between the plots. Four supplemental overhead lighting treatments were applied to the 16 plots in a Latin-Square design with 4 replications; every light treatment was applied in each row and each column of the square consisted of 16 plots. The supplemental light was provided by Pro 325e smart LED fixtures from LumiGrow (Emeryville, CA, USA). Treatment 1 (T1; control) was 16 h of red light (149 ± 6 µmol m^−2^ s^−1^; 06:00–22:00) and blue light (25 ± 1 µmol m^−2^ s^−1^; 06:00–22:00) followed by 8 h of darkness (22:00–06:00). Treatment 2 (T2) was a continuous (24 h) of red light (96 ± 3 µmol m^−2^ s^−1^) and blue light (17 ± 1 µmol m^−2^ s^−1^). Treatment 3 (T3) was 16 h of red light (151 ± 4 µmol m^−2^ s^−1^; 06:00–22:00) followed by 8 h of blue light (40 ± 3 µmol m^−2^ s^−1^; 22:00–06:00). Treatment 4 (T4) was 12 h of red light (210 ± 3 µmol m^−2^ s^−1^; 06:00–18:00) followed by 12 h of blue light (31 ± 2 µmol m^−2^ s^−1^; 18:00–06:00; Figure 1). Spectral compositions can be found in Figure 2 and were obtained using a spectroradiometer at night (Li−180, Li-COR Inc. Lincoln, NE, USA). T2 was used to minimize light fixture (capital cost), while T3 and T4 were selected based on our previous research on tomatoes that CL with 100% blue LED light less than 50 µmol m^−2^ s^−1^ does not cause any leaf chlorosis [4]. All supplemental light intensities were measured at a distance of 1 m from the light fixtures at 6 different locations with a 1 m long Li-COR line quantum sensor (Li-191R, Li-COR Inc., Lincoln, NE, USA). The total daily light integral (DLI) at 1 m from the supplemental lights was constant among all light treatments (~10.0 ± 0.1 mol m^−2^ d^−1^) and had similar percentages of red (86%) and blue (14%). Supplemental light treatments began on 1 November 2019. Throughout the experiment, supplemental lighting remained on regardless of ambient light levels to ensure all treatments received the same total DLI. The curtains were closed during cloudy days and at night to prevent treatment contamination. During sunny days, the curtains were opened to avoid the shading of natural light.

### 2.2. Growth Measurements

Plant height was measured 19 days into treatment (DIT) using a measuring tape. At 19, 75, and 103 DIT, the distance between the top of the plant and the 10th leaf was measured. Using this measurement, the internode length was determined. Furthermore, 19, 75, and 103 DIT, the 5th, 10th, and 15th leaf length and width, and SPAD readings were obtained using a chlorophyll meter (SPAD model 502, Konica Minolta, Osaka, Japan).

### 2.3. Leaf Gas Exchange: Day and Night Measurements

The 5th fully expanded leaf was placed in the chamber of a Li-COR 6400 (Li-COR Inc., Lincoln, NE, USA), which was fitted with a 2 cm × 3 cm clear top chamber. The leaf temperature was set to 25 °C with a relative humidity of 60–70% and a CO_2_ level held at 1000 µL L^−1^. Four leaves from separate plants under each treatment were used at 20 and 100 DIT for the day measurements and 19 and 98 DIT for the night measurements. Measurements taken during the day were performed on cloudy days to maximize the effect of supplemental lighting while minimizing the effect of natural light. Night measurements were performed after 22:30, which was during the subjective night period of all light treatments. This was done to assess how cucumber leaves growing under CL were able to utilize light during a period typically absent of ambient or supplemental radiation. Leaves were kept in the chamber until a steady-state photosynthesis rate was obtained, then the average from a 2-min period was taken.

### 2.4. Leaf Gas Exchange: Light Response Curves

The 5th fully expanded leaf was placed in the chamber of a Li-COR 6400, which was fitted with a 2 cm × 3 cm red/blue (88% R/12% B) LED Li-COR standard light source. The leaf temperature was set to 25 °C with a relative humidity of 60–70% and a CO_2_ level held at 1000 µL L^−1^. Three leaves from separate plants under each treatment were used at 20 and 99 DIT. Measurements were performed on cloudy days. Leaves were acclimated to high light intensity (1500 µmol m^−2^ s^−1^) until a steady-state photosynthetic rate was achieved. After the steady-state was achieved, light curves began at a high light intensity and decreased gradually following the procedure from Lanoue et al. [21]. At each light level, the photosynthetic rate was allowed to reach a steady-state then measurement was taken for that light level. Photosynthetic rates were plotted against the light intensity and fitted to a regression line following the equation y = y_o_ + a (1 − e^(−b*x)^) using SigmaPlot 10.0, where y is the photosynthetic rate at a given x, y_o_ is the calculated respiration rate, a is the photosynthetic maximum, b is the extinction coefficient of light capture, and x is the light intensity. A linear regression (y = mx + b) using the photosynthetic rates between the light levels of 0–100 µmol m^−2^ s^−1^ was used to calculate both the light compensation point (LCP) and quantum yield (QY) where y is the photosynthetic rate at a given x, m is the slope of the line (QY), x is the light intensity, and b is the photosynthetic rate calculated when y = 0.

### 2.5. Leaf Gas Exchange: CO_2_ Response Curves

The 5th fully expanded leaf was placed in the chamber of a Li-COR 6400, which was fitted with a 2 cm × 3 cm red/blue (88% R/12% B) LED Li-COR standard light source. The leaf temperature was set to 25 °C with a relative humidity of 60–70% and a light level of 1000 µmol m^−2^ s^−1^. Three leaves from separate plants under each treatment were used at 19 and 96 DIT. Leaves were acclimated to a light level of 1000 µmol m^−2^ s^−1^ and a CO_2_ level of 1000 µL L^−1^ prior to beginning the CO_2_ response curve. Measurements were performed on cloudy days. CO_2_ response curves began at the ambient CO_2_ concentration (1000 µL L^−1^) and reduced incrementally to 50 µL L^−1^. After the 50 µL L^−1^ measurement, the CO_2_ concentration was set to 1000 µL L^−1^ and was held steady until plant photosynthetic parameters returned to levels established during the beginning of the experiment. The CO_2_ level was then increased incrementally to 2000 µL L^−1^, at which point the CO_2_ response curve was terminated. At each specified CO_2_ concentration, the photosynthetic rate was allowed to reach a steady-state then one measurement was taken to produce values for that CO_2_ concentration. CO_2_ levels were as follows: 1000, 800, 600, 400, 200, 100, 50, 1000, 1000, 1250, 1500, and 2000 µL L^−1^. Photosynthetic rates were plotted against internal CO_2_ concentration (C_i_) and fitted to the FvCB model [22], and temperature corrected [23,24] to determine the maximum rate of photosynthesis under Rubisco-limited and RuBP-limited conditions.

### 2.6. Chlorophyll Fluorescence Imaging

Intact leaves were dark-adapted using aluminum foil for 10 min. After the dark adaptation period, leaflets were detached and immediately used for chlorophyll imaging using a closed FluorCam model FC 800-C with FluorCam v.7.0 software (FluorCam, Photon System Instruments, Brno, Czech Republic). The minimum fluorescence in a dark-adapted state (F_o_) was acquired during a dark-period of 5 s, after which an 800 ms saturating light pulse (2400 µmol m^−2^ s^−1^) from a blue LED (peak emission of 449 nm) was used to measure maximum fluorescence in a dark-adapted state (F_m_). From F_o_ and F_m_, the variable fluorescence in a dark-adapted state (F_v_) was calculated (F_v_ = F_m_ − F_o_), which was used to determine the maximum photosystem II (PSII) quantum yield (F_v_/F_m_). In general, the lower the value of F_v_/F_m_, the more severe the photo-inhibition and, thus, the leaf injury [17]. Typical values of F_v_/F_m_ indicating a healthy leaf are around 0.83 [17,25]. By calculating F_v_/F_m_ using chlorophyll fluorescence imaging, we are able to assess not only the prevalence of injury but also the spatial heterogeneity of F_v_/F_m_ from a leaflet. Eight leaflets from the 5th leaf of eight different plants were used for each light treatment when plants were 95 DIT.

### 2.7. Yield

Harvest began on 19 November 2019, 19 DIT. Mini-cucumbers were harvested 3 times per week when they had a diameter of approximately 2.5 cm. The harvested fruit was graded according to commercial grading standards (Ontario Ministry of Agriculture, Food and Rural Affairs, 2010). The fruit number and weight for marketable and unmarketable fruit were recorded. Mini-cucumbers were grouped into three harvest periods: early yield (19 November 2019–27 December 2019), middle yield (28 December 2019–7 February 2020), and late yield (8 February 2020–20 March 2020).

### 2.8. Economic Analysis

The calculation of on-peak, mid-peak, and off-peak electrical consumption was done as follows:

$$E=\left(\frac{\left(\left(Lu/Lm\right)P*h\right)}{1000}\right)*f$$

where *E* is the electrical consumption during a specific period over the course of the experiment (kWh), *Lu* is the light intensity used (µmol m^−2^ s^−1^), *Lm* is the maximum light intensity of the fixture at 1 m from the light (µmol m^−2^ s^−1^), *P* is the wattage of the light fixture (310 W), *h* is the hours the light was on during a specific period, and *f* is the number of fixtures.

The total power consumed during the 141 days is the sum of all on-peak, mid-peak, and off-peak power consumed. The total electricity cost was the sum of the products of the total kWh during each peak period and the average price during that same period as determined via IESO pricing [24]. Total electricity cost was then normalized on bases of m^2^, kg of production, and per fruit using the fixture density and production numbs, respectively.

### 2.9. Statistical Analysis

All statistical analyses were performed using SAS Studio 3.5. A Shapiro–Wilk test was used to test for normality with an alpha of 0.05 in every data set. Analysis of variance (ANOVA) was conducted on plant growth and yield data according to Latin-Square design with 4 replications to separate any variance caused by the row and column in the greenhouse plot layout. Suppose the main effect for the lighting treatments from ANOVA is significant (*p* < 0.05), then means comparisons between the different treatments were performed using a Tukey–Kramer adjustment test at *p* < 0.05. ANOVA on plant physiological parameters was analyzed according to a completely randomized design, and the means were separated using a Tukey–Kramer adjustment test at *p* < 0.05 if the lighting treatment effect is significant in the ANOVA.

## 3. Results

### 3.1. Plant Growth

Morphologically, cucumber plants grown under the various light treatments were virtually identical. Initial plant height measured at 19 DIT was similar between all treatments (Table 1). During all growth measurement time points, the internode length, 5th, 10th, and 15th leaf length and width were similar among all treatments (Table 1). Furthermore, leaf chlorophyll readings with the SPAD meter from the 5th (38–46), 10th (43–51), and 15th (43–55) leaves were similar among all treatments within the respective measurement period.

### 3.2. Day and Night Leaf Gas Exchanges

**Figure 3:**
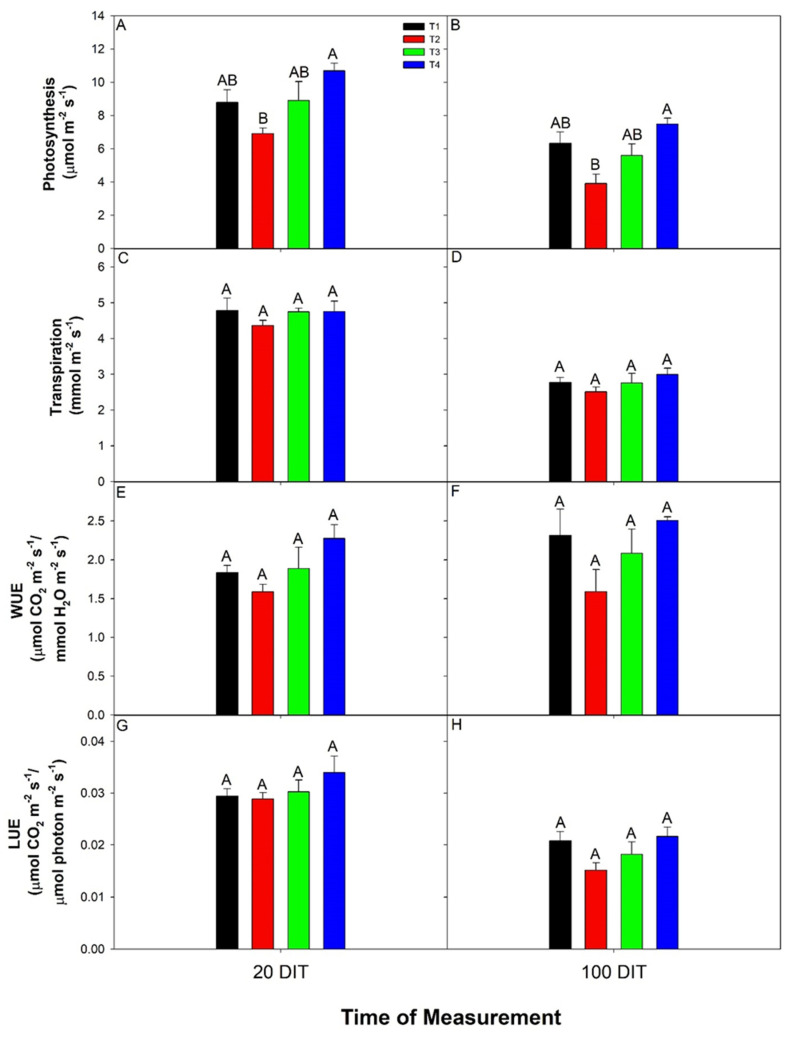
Photosynthesis (panels (**A**,**B**)), transpiration (panels (**C**,**D**)), water-use-efficiency (WUE; panels (**E**,**F**)), and light-use-efficiency (LUE; panels (**G**,**H**)) of the 5th leaf from mini-cucumber leaves grown under T1, T2, T3, and T4 lighting treatments at 20 DIT (days into treatment; panels (**A**,**C**,**E**,**G**)) or 100 DIT (panels (**B**,**D**,**F**,**H**)) during the day time. Measurements were performed using a Li-COR 6400 fitted with a clear top chamber on a cloudy day and thus represent the net carbon-exchange rate (NCER) driven mostly by the supplemental lighting. Error bars represent the standard error of the mean of n = 4. Letter groups (**A**,**B**) represent a significant difference within a panel between the lighting treatments at a specific data collection date at *p* < 0.05.

At both 20 and 100 DIT cucumber plants grown under T4 produced the highest daytime photosynthetic rate, while plants grown under T2 produced the lowest (Figure 3A,B). This is because T4 had the highest, while T2 had the lowest supplemental light intensity during the daytime. Leaf water gas exchanges were similar under all treatments indicating that the daytime water gas exchanges were not affected by CL (Figure 3C–F). Due to the different supplemental light intensities used between the treatments, light-use efficiency (LUE) was calculated to normalize the photosynthetic rate based on the incoming radiation. Upon normalization, daytime LUE values were similar among all treatments at both 20 and 100 DIT (Figure 3G,H).

**Figure 4:**
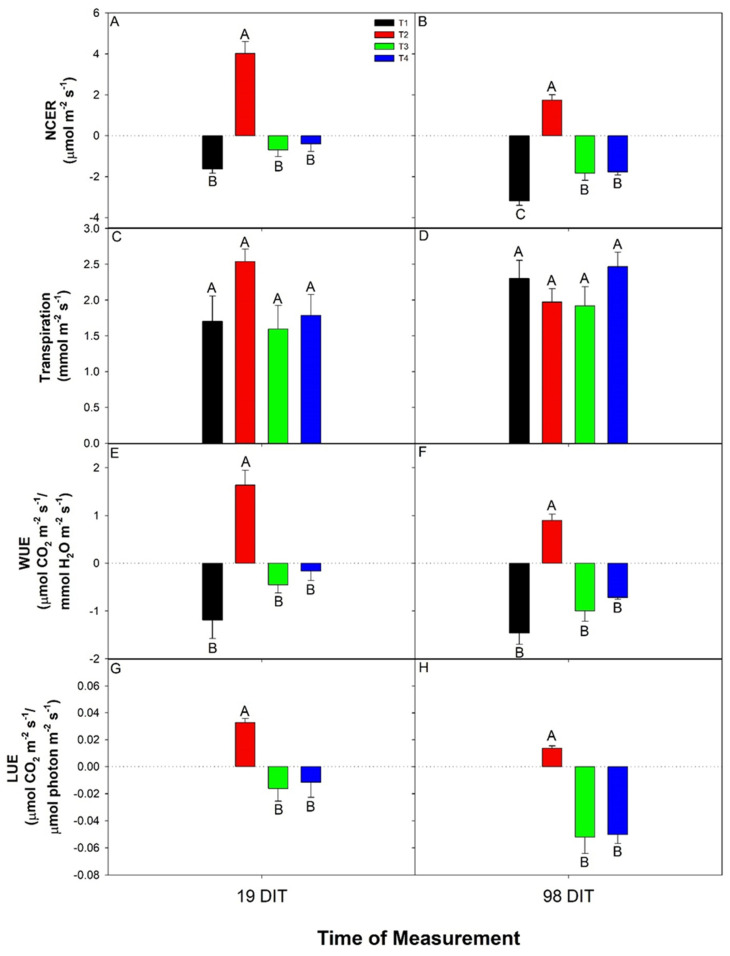
Net carbon-exchange rate (NCER; panels (**A**,**B**)), transpiration (panels (**C**,**D**)), water-use-efficiency (WUE; panels (**E**,**F**)), and light-use-efficiency (LUE; panels (**G**,**H**)) of the 5th leaf from mini-cucumber leaves grown under T1, T2, T3, and T4 lighting treatments at 19 DIT (days into treatment; panels (**A**,**C**,**E**,**G**)) or 98 DIT (panels (**B**,**D**,**F**,**H**)) during the nighttime. Measurements were performed using a Li-COR 6400 fitted with a clear top chamber and thus represent the NCER driven by the supplemental lighting. Error bars represent the standard error of the mean of n = 4. Letter groups (**A**,**B**) represent a significant difference within a panel between the lighting treatments at a specific data collection date at *p* < 0.05.

During the nighttime, at 19 and 98 DIT, the net carbon-exchange rate (NCER) of cucumbers was assessed during a period normally absent of radiation. At both 19 and 98 DIT, plants grown under T2 produced positive NCERs indicating photosynthesis and the assimilation of CO_2,_ while all other treatments produced negative NCERs typical of respiration during the night period (Figure 4A,B). In addition, at 98 DIT, the nighttime NCER from both T3 and T4 were higher than that from T1, indicating a lower respiration rate and less loss of carbon (Figure 4B).

At both 19 and 98 DIT, the transpiration rates from all treatments were similar (Figure 4C,D). Treatment 2 (T2) produced the highest WUE among all light treatments at both 19 and 98 DIT (Figure 4E,F). Due to the negative NCER rate produced under T1, T3, and T4, cucumber leaves were observed to have a negative WUE, indicating that both CO_2_ and H_2_O were leaving the leaf simultaneously. Although the light was being provided to the plants during the subjective night period under T3 and T4, respiration rates were still observed, which resulted in negative LUEs indicating that the photosynthetic rates were not high enough to compensate for dark respiration (Figure 4G,H). Thus, leaves under T2 had the highest LUE at both 19 and 98 DIT (Figure 4G,H). No LUE was calculated for T1 as the light intensity during the night period was 0, leaving the calculation of LUE (NCER/light intensity) non-resultant.

### 3.3. Leaf Light and CO_2_ Response Curves

Leaf light response curves were performed on cucumber leaves grown under all light treatments at 20 and 99 DIT. This allowed for the analysis of leaf photosynthetic machinery during a range of light intensities from light saturating to light limiting. Upon analysis, the respiration rate, light compensation point (LCP), quantum yield (QY) and maximum photosynthetic rate (Pn_max_) were similar among all treatments at each measurement date (Table 2).

Both the maximum rate of Rubisco carboxylase activity (V_cmax_) and the maximum rate of photosynthetic electron transport (J_max_) give insight into the carbon assimilation process, indicating the maximum rate of RuBP regeneration and the maximum rate of electron transport, respectively. Thus, similar to values associated with photosynthetic light response curves, V_cmax_ and J_max_ give an indication of a leaf’s function under certain growth conditions. At 19 DIT, cucumber leaves grown under T2 produced the lowest V_cmax_ and J_max_ compared to the other light treatments (Table 3). At 96 DIT, leaves grown under both T2 and T3 produced lower V_cmax_ rates than did those grown under T1 (Table 3). Similar to leaves analyzed at 19 DIT, leaves grown under T2 had the lowest J_max_ rate at 96 DIT (Table 3). The J_max_:V_cmax_ was similar among all treatments at both 19 and 96 DIT measurements indicating that neither the RuBP regeneration rate nor the rate of electron transport was preferentially affected by the light treatments (Table 3).

### 3.4. Leaf Chlorophyll Fluorescence

**Figure 5:**
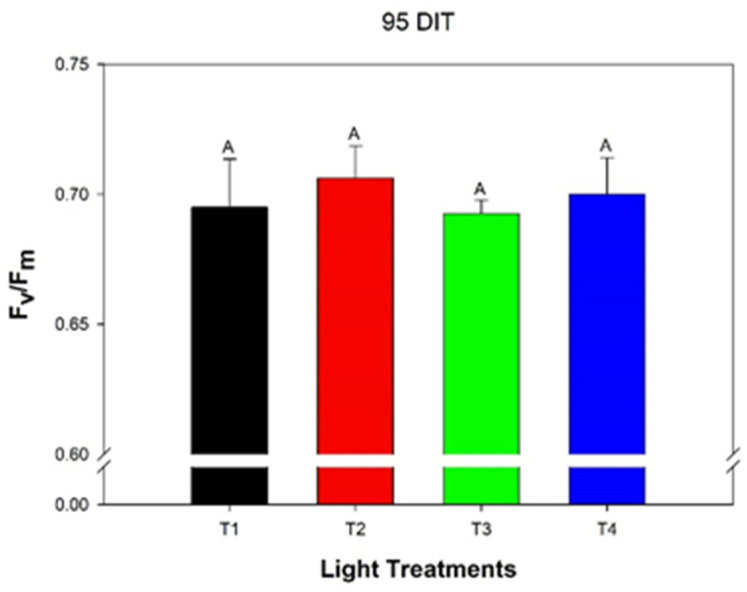
Quantum efficiency of PSII (F_v_/F_m_) of the 5th leaf from mini-cucumbers grown under either T1, T2, T3, or T4 light treatment at 95 days into treatment (DIT). Error bars represent the standard error of the mean of n = 8. Letter groups represent a significant difference between the lighting treatments at *p* < 0.05.

**Figure 6:**
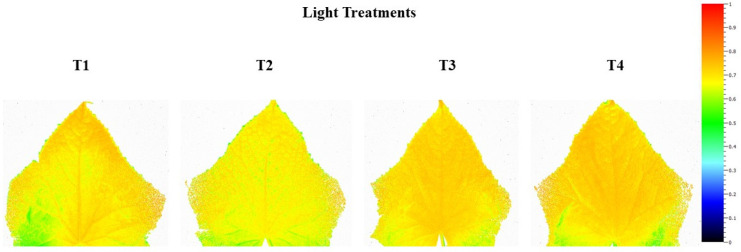
Spatial response of quantum efficiency (F_v_/F_m_) from the 5th leaf of mini-cucumbers grown under either T1, T2, T3, or T4 light treatment measured at 95 days into treatment (DIT).

Leaves under all treatments produced similar F_v_/F_m_ values indicating no effect of light treatment on photo-inhibition (Figure 5). Spatial variations in F_v_/F_m_ were also obtained in order to see if the areas of leaves were affected differently based on the light treatment (Figure 6). At the base and edges (Figure 6) of cucumber leaves, the resolution of the image began to deteriorate, which is a technical function of the equipment and the large size of the leaf used, which we believe led to the lower than optimal values. No obvious difference in spatial F_v_/F_m_ values between the treatments was observed. Lower than expected F_v_/F_m_ values were obtained and are most likely the results of spatial resolution during imaging.

### 3.5. Fruit Production

During all production periods, there was no difference in fruit number, total fruit weight, or average fruit weight among the light treatments (Table 4). The difference in fruit yield between T1 (control) and T2 was less than 0.5%. These results indicate that CL lighting strategies did not compromise fruit production regardless of the natural solar radiation.

### 3.6. Economical Analysis

Extended photoperiods can allow for lower light intensities to be used while still achieving the desired DLI. Therefore, the use of CL can reduce the lighting capital costs. In the case of T2, the light intensity/capital costs can be reduced by 1/3 in comparison to T1 (control). Furthermore, in Ontario, Canada, electricity prices vary depending on the period of time it is used. Periods of time such as nighttime typically have lower electricity costs than daytime. For this reason, the utilization of supplemental CL LED strategies such as T2 could reduce electricity cost by up to 10% compared to the traditional 16 h photoperiod (T1) without compromising fruit yield and quality (Table 5).

## 4. Discussion

The implementation of supplemental lighting during greenhouse production has increased the growth rates and yield of cucumbers [11,12,27]. The use of CL is the pinnacle utilization of supplemental lighting as it provides continuous 24 h of radiation to the crop. Theoretically, the use of CL provides constant energy for carbon assimilation, meaning larger biomass accumulation and yield [5]. Furthermore, the implementation of a supplemental CL strategy can have a positive economic implication as well if no CL-injury occurs, such is the case in this study (Table 5). While the most advanced double-ended 1000 W HPS fixtures have a photosynthetic photon efficacy (PPE) of 1.7 µmol J^−1^, the current LEDs have a PPE at 2.1 to 3.1 µmol J^−1^ with a theoretical maximum of around 4.7 µmol J^−1^, depending on the wavelength [28]. Compared to HPS lighting, the utilization of energy-efficient LEDs (3.0 µmol J^−1^) can drastically increase energy efficiency and decrease electrical energy use by 70%. However, the high light fixture costs have been the largest barrier impacting the adoption of energy-efficient LEDs in greenhouse crop production [29]. Today, a typical HPS fixture costs between $200–$400 Canadian dollars (CAD), while a comparable LED fixture can cost between $1000–$2000 CAD, depending on the company and type. Under the current market conditions, the LED light fixture costs to provide 210 µmol m^−2^ s^−1^ PAR (photosynthetically active radiation; recommended light intensity for greenhouse tomato and cucumber production) for 1 ha of the greenhouse is about $2.5 million CAD, while it is only about $350,000 CAD for the double-ended 1000 W HPS fixtures. The utilization of CL as a supplemental lighting strategy allows for lower light intensities, such as T2, which can reduce the number of LED fixtures/light fixture costs by 1/3 ($800,000 CAD in savings), which will facilitate the adoption of energy-efficient LEDs in greenhouse crop production. The discovery of CL strategies, which do not cause injury to fruiting vegetables widely grown in Canada and worldwide, can have extensive economic benefits while sustaining high yields.

The need to achieve target DLI with overall low light intensities is facilitated by extending the lighting to the whole night (re-allocation of some supplemental light from the day to the night). In some places, such as Ontario, Canada, electricity prices are reduced during the night periods (~50%), furthering the economic savings of CL compared to conventional supplemental lighting. Utilizing lighting at night can also supplement heating requirements during that period due to the heat output from the fixture. This can lead to a more uniform microclimate for plants, an underappreciated factor dictating plant growth [30]. Taken together, the use of CL is suitable for mini-cucumber production leading to the same yield parameters as a conventional supplemental lighting photoperiod and can also have positive economic implications.

Previous research related to CL and cucumber production used high-pressure mercury lamps as a lighting source in greenhouses or within growth chambers [15,16]. It was determined that as early as four weeks into the growth period, leaf chlorosis was observed [15]. Furthermore, at 9 weeks into the growth period, fruit yield was drastically reduced by CL compared to an 18 h photoperiod [15]. Thus, our study represents the first full greenhouse production cycle of mini-cucumbers grown under CL in which no CL-injury was observed, and yield parameters from CL treatments were similar to a traditional 16 h photoperiod (Figure 5 and Table 4). Of note, each crop cycle of mini-cucumbers is only about four months in commercial production, whereas our greenhouse was close to five months.

Treatment 2 (T2) provided approximately 115 µmol m^−2^ s^−1^ of red and blue light for 24 h, a light intensity well above the light compensation point. This treatment was unique among the four used as it was the only treatment which did not employ a decrease in light intensity or a change in the light spectrum. It was also unique as it was the only treatment which produced a photosynthetic rate (net carbon gain) during the night periods for measurements taken at both 19 and 98 DIT (Figure 4A,B). A high nighttime light intensity invoking a photosynthetic rate typically increases the photo-oxidative stress leading to CL-injury if no interventions (i.e., temperature dip, decreased light intensity, or change in spectral quality) are used [2]. However, in our study, no CL-injury was observed from T2. Similar J_max_:V_cmax_ values between light treatments (Table 3) indicate that photosynthesis under CL is not preferentially inhibited by either electron transport or RuBP regeneration, as was the case in Haque et al. [31]. Notably, V_cmax_ and J_max_ from T2 were decreased at both 19 and 96 DIT measurements compared to the other treatments (Table 3). The decrease in these two values could be explained by a decrease in nitrogen content within the leaf [32]. The constant high photo-oxidative pressure from T2 could cause a re-allocation of nitrogen within the plant to help mitigate damage via further production of scavenger proteins. The V_cmax_ and J_max_ were measured under 1000 µmol m^−2^ s^−1^ of light, which was not a light intensity that occurred in this greenhouse trial or representative of winter production in Ontario or other northern regions. Therefore, the plant growth and fruit production were not affected despite the decrease in V_cmax_ and J_max._

One hypothesis as to why no CL-injury was observed is that the total DLI was below the critical point for cucumbers. During the winter months in southern Ontario, solar DLI is typically below 10 mol m^−2^ d^−1^ [33,34]. During the production stage of a cucumber crop, plants have been observed to grow adequately in up to 25 mol m^−2^ d^−1^ [35] above which photo-oxidative stress is observed. Over such a DLI, the photo-protective mechanisms are not able to handle the photo-oxidative stress and thus light utilization, plant growth, and yield are decreased [36]. In this study, the supplementary CL treatments used in addition to the natural solar DLI likely did not surpass the 25 mol m^−2^ d^−1^ DLI threshold of cucumbers during the production period, avoiding CL-injury even though T2 was observed to produce continuous (24 h) photosynthesis.

However, some studies using light intensities well below the threshold limit of cucumbers have still observed injury during a CL strategy [16]. Shibaeva and Markoskaya [16] observed a significantly lower F_v_/F_m_ from plants grown under 160 µmol m^−2^ s^−1^ for 24 h as opposed to those grown at the same light intensity for 16 h. At such a light intensity, the DLI (~13.8 mol m^−2^ d^−1^) was still well below the proposed 25 mol m^−2^ d^−1^ threshold for cucumbers [35]. There are two major differences between the Shibaeva and Markoskaya [16] study and ours which may explain why no CL-injury was observed. First, their study was done in a growth chamber which was absent of natural solar radiation. Second, our study utilized wavelength-specific LEDs (red and blue), whereas Shibaeva and Markoskaya [16] utilized a high-pressure mercury light source. The high-pressure mercury lamp emits much more thermal radiation than LEDs which may lead to high top canopy temperature and stress. It has been shown that tomatoes grown under CL with a constant temperature had developed CL related injury [31]. However, when exposed to a decrease in nighttime temperature, tomato plants grown under CL were not observed to have typical traits associated with CL-injury [31]. Thus, due to the reduction in radiant heat emitted from LED fixtures (55% of output energy for HPS compared to 27% for LED), LEDs can reduce the heat load enough during the night to mitigate CL-injury, allowing for the use of such production strategies.

The absence of natural solar radiation does not only reduce the overall light intensity the plants are exposed to but also eliminates the gradual rise and fall of the light intensity and spectral changes (such as red to far-red ratio) typically associated with sunrise and sunset. Typically, growth under drastically altered photoperiods (i.e., too long or too short) leading to circadian asynchrony has negative consequences on plant biological functions such as biomass accumulation, flowering, and general metabolism [7,37,38,39]. Thus, a hypothesis may be made that the addition of the supplemental CL to the natural solar photoperiod/spectral change may allow plants to maintain their entrained circadian rhythms. However, many studies have used supplemental CL during the greenhouse production of cucumber and tomatoes and still observed CL-injury [15,40,41]. One noticeable difference between those studies and our current study was the type of supplemental lighting used. While previous studies utilized broad spectrum HID (high-intensity discharge; HPS or high-pressure mercury) as a CL source, our study used wavelength-specific LEDs. Thus, the results presented here and in previous studies [4] cannot solely be attributed to the natural solar intensity and must have an interaction with the properties of the CL sources and the spectral changes over a day cycle (i.e., thermal properties or spectral quality).

The use of wavelength-specific lighting has been proposed to play a key role in the susceptibility of plants to CL-injury. Velez-Ramirez et al. [10] determined that phytochrome A (PHY A) may play a role in reducing the incidence of CL-injury in tomatoes. It was determined that the overexpression of PHY A led to reduced injury via chlorophyll fluorescence imaging [10]. PHY A is a photoreceptor controlled by the ratio of red to far-red light, turning the light signals into biological functions where the absorption of red light converts PHY A into its active form [42,43]. Furthermore, PHY A has been shown to retain some of its function during low levels of blue light [43]. One of the functions of PHY A is the promotion of anthocyanin biosynthesis during times of high red light and low blue light, which can aid in the reduction of photo-oxidative damage [44,45]. It is hypothesized that the use of wavelength-specific LEDs emitting higher components of red light during CL strategies allowed for higher tolerance to CL, thus alleviating injury. Similarly, an alleviation of CL related injury was also observed in an experiment on tomato, which used a red and blue alternating CL strategy [4] as well as when tomatoes were grown under 23 h of supplemental red light (Data unpublished).

In this study, all CL supplemental treatments were observed to have no CL-injury over the course of the production period. In a study using similar lighting treatments, tomato plants grown under similar conditions to T2 showed CL-injury (Data unpublished). These two important greenhouse crops were observed to have two different responses to very similar CL lighting treatments. Much research has been performed on tomatoes with respect to CL causing injury. One prevailing hypothesis is that the over-accumulation of sucrose and starch has negative impacts on F_v_/F_m_, a common indicator used for CL-injury [8]. The major difference between cucumber and tomato are their carbohydrate metabolism, specifically their carbon export process. In tomatoes, sucrose is the primary phloem mobile carbohydrate, whereas, in cucumbers, raffinose and stachyose are the main carbohydrates exported [46,47]. Moreover, tomatoes use an apoplastic phloem loading mechanism, whereas cucumbers use a symplastic polymer trap mechanism. These two phloem loading mechanisms have inherent differences. Namely, apoplastic phloem loading is a complicated pathway involving multiple cell lines, enzymes, and transporters, which all act as potential points of regulation [47,48]. Symplastic phloem loading is much more rudimentary, with minimal points of enzymatic regulation [46]. Due to the form of phloem loading and the production of auxiliary sugars such as raffinose and stachyose, cucumbers are known to be rapid exporters of recently fixed carbon, minimizing carbohydrate accumulation (i.e., starch) in the leaf [49]. When discussing carbohydrate over-accumulation and its potential implications in CL-injury, the rate of sugar efflux from the leaf must not be overlooked. Thus, we propose that due, in part, to the rapid rate of carbon export known to occur in cucumbers, the over-accumulation of sugars may be curtailed, making cucumbers more tolerant to CL than tomatoes.

## 5. Conclusions

In summary, the results presented in this study indicate that mini-cucumber plants can be grown for a full production cycle under CL with LEDs without any photoperiod related injury. All morphological, physiological, and yield parameters measured were similar among all treatments tested. Overall, mini-cucumber production was similar under three CL strategies compared to a conventional photoperiod while having a positive economic impact due to the significant reduction in light fixture costs and the redistribution of electrical usage from day to night, leading to lower electricity costs.

## Figures and Tables

**Figure 1:**
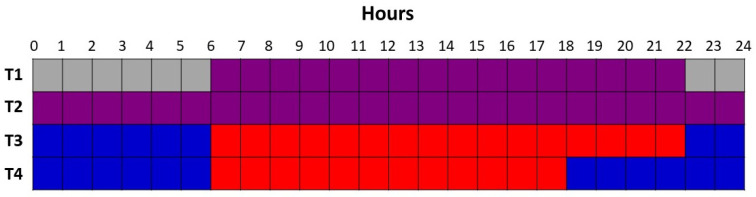
Light treatment schematic showing the period of time for which each light treatment was on and the spectrum of the light emitted during that period. Gray shading represents darkness (nighttime), purple shading represents red + blue light from light-emitting diodes (LEDs), blue shading represents blue light from LEDs, and red shading represents red light from LEDs.

**Table 1:** Major growth characteristics of cucumber plants grown under T1, T2, T3, or T4 at 19, 75, and 103 days into treatment (DIT). The internode length was calculated by measuring from the tip of the plant to the 10th leaf (distance/10). Notably, at 75 and 103 DIT, total plant height was not measured due to physical constraints. All averages ± the standard error represent 6 plants. Letter groups represent a significant difference at a measurement time between the lighting treatments at *p* < 0.05.

Treatment	Plant Height (cm)	Internode Length (cm)	Length 5th Leaf (cm)	Width 5th Leaf (cm)	Length 10th Leaf (cm)	Width 10th Leaf (cm)	Length 15th Leaf (cm)	Width 15th Leaf (cm)
**19 DIT**								
**T1**	200 ± 3^A^	9.3 ± 0.2^A^	18.7 ± 0.3^A^	19.2 ± 0.6^A^	24.3 ± 0.6^A^	26.2 ± 0.7^A^	27.2 ± 0.7^A^	30.3 ± 0.6^A^
**T2**	205 ± 5^A^	9.4 ± 0.3^A^	18.7 ± 0.3^A^	19.0 ± 0.3^A^	24.2 ± 0.7^A^	25.7 ± 0.9^A^	27.0 ± 1.1^A^	31.2 ± 1.1^A^
**T3**	200 ± 3^A^	9.1 ± 0.1^A^	17.7 ± 0.3^A^	17.7 ± 0.5^A^	23.3 ± 0.9^A^	23.7 ± 0.8^A^	28.2 ± 0.8^A^	32.0 ± 1.2^A^
**T4**	193 ± 5^A^	8.9 ± 0.3^A^	19.3 ± 0.6^A^	19.3 ± 0.7^A^	25.0 ± 0.5^A^	26.3 ± 1.0^A^	26.2 ± 0.7^A^	31.7 ± 0.9^A^
**75 DIT**								
**T1**	N/A	9.1 ± 0.2^A^	19.7 ± 0.3^A^	23.7 ± 0.3^A^	24.2 ± 0.3^A^	29.5 ± 0.6^A^	27.0 ± 0.5^A^	31.3 ± 0.6^A^
**T2**	N/A	9.3 ± 0.1^A^	18.8 ± 0.6^A^	23.0 ± 0.6^A^	23.3 ± 0.5^A^	28.3 ± 0.8^A^	26.0 ± 0.7^A^	30.7 ± 0.7^A^
**T3**	N/A	9.4 ± 0.1^A^	19.5 ± 0.4^A^	23.3 ± 0.3^A^	23.5 ± 0.7^A^	27.7 ± 1.1^A^	25.7 ± 0.8^A^	30.5 ± 0.8^A^
**T4**	N/A	9.3 ± 0.2^A^	19.5 ± 0.6^A^	24.0 ± 0.8^A^	24.5 ± 0.4^A^	28.8 ± 0.7^A^	26.7 ± 0.8^A^	31.0 ± 1.2^A^
**103 DIT**								
**T1**	N/A	8.3 ± 0.2^A^	17.5 ± 0.4^A^	20.8 ± 0.4^A^	25.8 ± 0.5^A^	30.2 ± 0.9^A^	27.8 ± 0.4^A^	33.0 ± 0.8^A^
**T2**	N/A	7.9 ± 0.2^A^	18.3 ± 0.4^A^	20.8 ± 0.3^A^	25.3 ± 0.3^A^	30.5 ± 0.9^A^	26.3 ± 0.5^A^	32.3 ± 0.2^A^
**T3**	N/A	8.0 ± 0.2^A^	17.0 ± 0.4^A^	20.5 ± 0.5^A^	24.7 ± 0.7^A^	29.3 ± 0.8^A^	27.7 ± 0.5^A^	31.2 ± 0.7^A^
**T4**	N/A	8.0 ± 0.1^A^	17.2 ± 0.2^A^	20.5 ± 0.2^A^	25.7 ± 0.7^A^	30.5 ± 0.8^A^	27.3 ± 0.6^A^	33.5 ± 1.0^A^

**Table 2:** Summary of the major physiological traits as determined by leaf light response curves (Supplementary Figure S1) from mini-cucumbers grown under T1, T2, T3, or T4 at 20 and 99 days into treatment (DIT). Respiration values were the averages of net carbon exchange rate (NCER) when the light level was 0 µmol m^−2^ s^−1^. The light compensation point (LCP) and quantum yield (QY) were calculated from a regression line (y = mx + b) fitted to the values between the photosynthetically active radiation (PAR) values of 0–100 µmol m^−2^ s^−1^. The photosynthetic maximum (Pn_max_) was calculated from y = y_o_ + a (1 − e^(−b*x)^). Values ± the standard error of the mean are representative of n = 3. Within each parameter and measurement date, letter groups represent a statistical difference as determined by a one-way ANOVA with a Tukey–Kramer adjustment (*p* < 0.05).

Light Treatment	Respiration (µmol CO_2_ m^−2^ s^−1^)	LCP (µmol m^−2^ s^−1^)	QY (µmol CO_2_ m^−2^ s^−1^/ µmol m^−2^ s^−1^)	Pn_max_ (µmol CO_2_ m^−2^ s^−1^)
**20 DIT**				
**T1**	−1.73 ± 0.49^A^	36.94 ± 8.56^A^	0.046 ± 0.001^A^	25.98 ± 1.34^A^
**T2**	−2.65 ± 0.21^A^	51.48 ± 4.22^A^	0.051 ± 0.004^A^	26.12 ± 0.72^A^
**T3**	−2.18 ± 0.60^A^	38.14 ± 8.70^A^	0.055 ± 0.001^A^	26.41 ± 1.73^A^
**T4**	−1.92 ± 0.10^A^	35.13 ± 3.64^A^	0.051 ± 0.004^A^	25.80 ± 1.21^A^
**99 DIT**				
**T1**	−2.13 ± 0.10^A^	46.53 ± 0.10^A^	0.042 ± 0.002^A^	17.69 ± 0.60^A^
**T2**	−2.76 ± 0.23^A^	57.88 ± 8.00^A^	0.049 ± 0.005^A^	16.23 ± 1.45^A^
**T3**	−2.59 ± 0.06^A^	55.39 ± 3.08^A^	0.045 ± 0.002^A^	17.37 ± 0.89^A^
**T4**	−2.41 ± 0.04^A^	50.87 ± 1.73^A^	0.047 ± 0.002^A^	18.01 ± 0.48^A^

**Table 3:** Summary of the major physiological traits as determined by leaf CO_2_ response curves (Supplementary Figure S2) from mini-cucumbers grown under T1, T2, T3, or T4 at 19 and 96 days into treatment (DIT). V_cmax_ is the maximum rate of Rubisco carboxylation, and J_max_ is the maximum rate of electron transport. Values ± the standard error of the mean are representative of n = 3. Within each parameter and measurement date, letter groups (A–C) represent a statistical difference as determined by a one -way ANOVA with a Tukey–Kramer adjustment (*p* < 0.05).

Light Treatment	V_cmax_ (µmol CO_2_ m^−2^ s^−1^)	J_max_ (µmol e^-^ m^−2^ s^−1^)	J_max_:V_cmax_
**19 DIT**			
**T1**	40.51 ± 0.92^AB^	119.17 ± 3.44^A^	2.94 ± 0.03^A^
**T2**	36.89 ± 1.35^B^	107.53 ± 4.07^B^	2.91 ± 0.02^A^
**T3**	40.05 ± 2.06^AB^	116.04 ± 5.15^AB^	2.90 ± 0.03^A^
**T4**	42.89 ± 1.63^A^	125.44 ± 5.42^A^	2.92 ± 0.02^A^
**96 DIT**			
**T1**	36.93 ± 0.61^A^	108.15 ± 1.83^A^	2.93 ± 0.003^A^
**T2**	24.63 ± 1.72^C^	71.08 ± 5.29^B^	2.88 ± 0.02^A^
**T3**	30.84 ± 0.63^B^	89.12 ± 1.69^AB^	2.89 ± 0.01^A^
**T4**	36.51 ± 1.56^AB^	106.00 ± 5.61^A^	2.90 ± 0.04^A^

**Table 4:** Major yield parameters of mini-cucumbers grown under either T1, T2, T3, or T4 light.

Yield Period	Light Treatment	Fruit Number (m^−2^)	Total Fruit Weight (kg m^−2^)	Average Fruit Weight (g Fruit^−1^)
**19 November 2019–27 December 2019**	T1	149^A^	12.02^A^	80.67^A^
	T2	145^A^	11.78^A^	81.24^A^
	T3	147^A^	11.92^A^	81.09^A^
	T4	148^A^	11.82^A^	79.86^A^
**28 December 2019–7 February 2020**	T1	149^A^	11.61^A^	77.92^A^
	T2	149^A^	11.52^A^	77.32^A^
	T3	147^A^	11.38^A^	77.41^A^
	T4	152^A^	11.64^A^	76.58^A^
**8 February 2020–20 March 2020**	T1	149^A^	12.87^A^	86.38^A^
	T2	155^A^	13.03^A^	84.06^A^
	T3	155^A^	13.19^A^	85.10^A^
	T4	157^A^	13.29^A^	84.65^A^
**19 November 2019–20 March 2020**	T1	448^A^	36.50^A^	81.50^A^
	T2	449^A^	36.34^A^	80.94^A^
	T3	448^A^	36.49^A^	81.45^A^
	T4	456^A^	36.76^A^	80.61^A^

Letter groups represent a significant difference between the lighting treatments at *p* < 0.05.

**Table 5:** Electrical energy consumption, cost, and yield parameters for mini-cucumbers grown under different light treatments during production between 1 November 2019, and 20 March 2020.

Light Treatment	T1	T2	T3	T4
Total electrical consumption (kWh)	6184	6184	6184	6184
On-peak electrical consumption (kWh) ^Z^	2319	1546	1988	2282
Mid-peak electrical consumption (kWh) ^Y^	2319	1546	1988	2650
Off-peak electrical consumption (kWh) ^X^	1546	3092	2209	1251
Total electricity cost	$105.70	$95.94	$101.52	$106.68
Total electricity cost per m^2^ of grow area	$4.52	$4.10	$4.34	$4.56
Total electricity cost per kg of production ^v^	¢12.38	¢11.28	¢11.89	¢12.40
Total electricity cost per fruit ^W^	¢1.01	¢0.91	¢0.97	¢1.00

^Z^ On-peak hours = 07:00–11:00 and 17:00–19:00. ^Y^ Mid-peak hours = 11:00–17:00. ^X^ Off-peak hours = 19:00–07:00. ^W^ Hourly prices were based on the 5 month average within each electrical period from IESO [26]. ^V^ Yield values are taken from Table 4.

## Data Availability

All data is contained within the article or supplementary material.

## Supplementary Materials

The following are available online at https://www.mdpi.com/2223-7747/10/2/378/s1, Supplementary Figures S1 and S2.
