# Supplementary material for: Continuous Light Does Not Compromise Growth and Yield in Mini-Cucumber Greenhouse Production with Supplemental LED Light
Source: Plants (Basel). 2021 Feb 17;10(2):378. doi: 10.3390/plants10020378 (PMC7921946; doi:10.3390/plants10020378)

Supplementary Figures

Supplementary Figure 1: Photosynthetic light response curves of mini-cucumber leaves grown under either T1, T2, T3, or T4 at 20 (Panel A) or 99 DIT (Panel B) as determined using a Li-COR 6400 with a red/blue standard Li-COR light source. T1 (control) was 16h of red light (149±6 µmol m^{-2} s^{-1}; 06:00-22:00) and blue light (25±1 µmol m^{-2} s^{-1}; 06:00-22:00) followed by 8h of darkness (22:00-06:00). T2 was a continuous (24h) of red light (96±3 µmol m^{-2} s^{-1}) and blue light (17±1 µmol m^{-2} s^{-1}). T3 was 16h of red light (151±4 µmol m^{-2} s^{-1}; 06:00-22:00) followed by 8h of blue light (40±3 µmol m^{-2} s^{-1}; 22:00-06:00). T4 was 12h of red light (210±3 µmol m^{-2} s^{-1}; 06:00-18:00) followed by 12h of blue light (31±2 µmol m^{-2} s^{-1}; 18:00-06:00). Measurements were made at a CO_2 level of 1000 µL L^{-1}, leaf temperature of 25ºC, and relative humidity of 60-70%. Regressions lines were fit to $y=y_o+a(1-e^{(-b*x)})$ for each lighting treatment. Inserts A' and B' are magnification of 0-100 µmol m^{-2} s^{-1} PAR regions fit to the regression line y=mx+b.

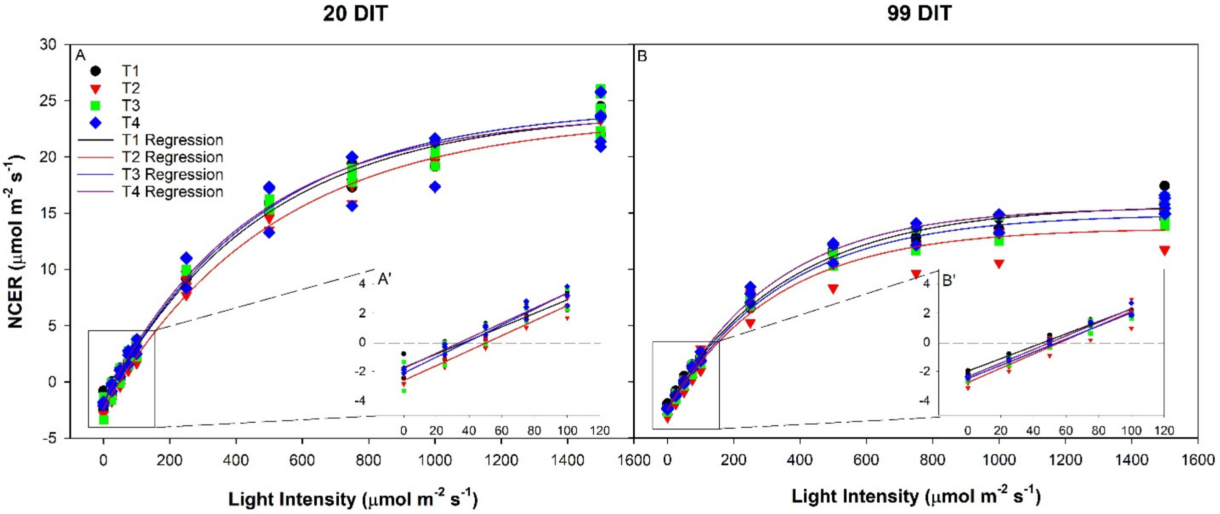

Supplementary Figure 2: Photosynthetic CO_2 response curves of leaves grown under either T1, T2, T3, or T4 at 19 or 96 DIT as determined using a Li-COR 6400 with a red/blue standard Li-COR light source. T1 (control) was 16h of red light (149±6 µmol m^{-2} s^{-1}; 06:00-22:00) and blue light (25±1 µmol m^{-2} s^{-1}; 06:00-22:00) followed by 8h of darkness (22:00-06:00). T2 was a continuous (24h) of red light (96±3 µmol m^{-2} s^{-1}) and blue light (17±1 µmol m^{-2} s^{-1}). T3 was 16h of red light (151±4 µmol m^{-2} s^{-1}; 06:00-22:00) followed by 8h of blue light (40±3 µmol m^{-2} s^{-1};

22:00-06:00). T4 was 12h of red light (210±3 µmol m^{-2} s^{-1}; 06:00-18:00) followed by 12h of blue light (31±2 µmol m^{-2} s^{-1}; 18:00-06:00). Measurements were made at a light level of 1000 µmol m^{-2} s^{-1}, temperature of 25ºC, and relative humidity of 60-70%. Rubisco and RuBP limited fit lines were determined using temperature corrections from McMurtrie and Wang (1993) and Bernacchi et al., (2001).

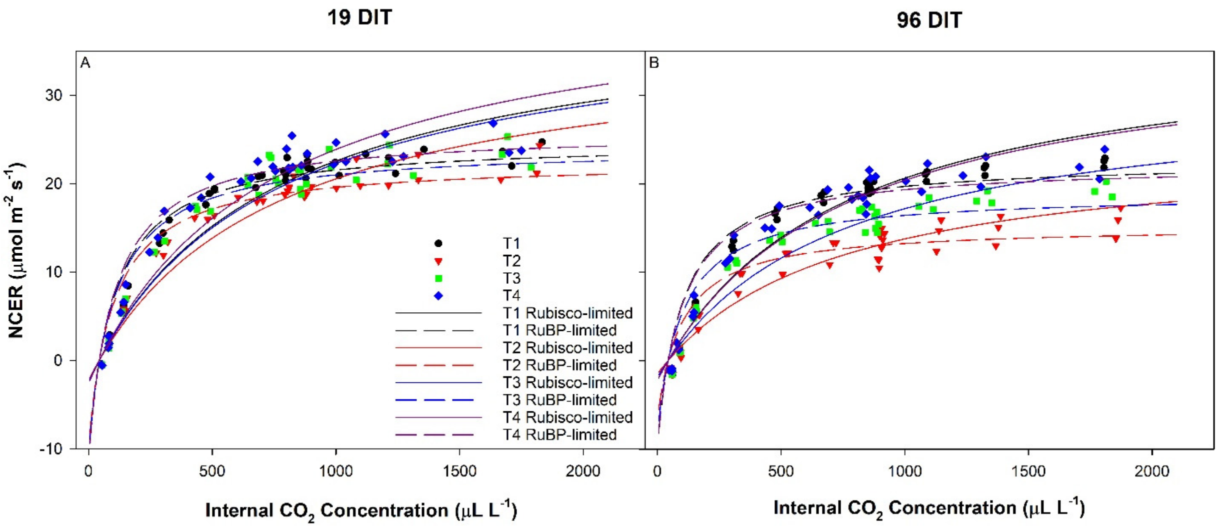

Supplement: Supplementary file 1 [file plants-10-00378-s001.pdf]
